# Mistletoe Extracts from Different Host Trees Disparately Inhibit Bladder Cancer Cell Growth and Proliferation

**DOI:** 10.3390/cancers15194849

**Published:** 2023-10-04

**Authors:** Eva Juengel, Jochen Rutz, Moritz Meiborg, Sascha D. Markowitsch, Sebastian Maxeiner, Timothy Grein, Anita Thomas, Felix K.-H. Chun, Axel Haferkamp, Igor Tsaur, Olesya Vakhrusheva, Roman A. Blaheta

**Affiliations:** 1 Department of Urology and Pediatric Urology, University Medical Center Mainz, 55131 Mainz, Germany; eva.juengel@unimedizin-mainz.de (E.J.); sascha.markowitsch@unimedizin-mainz.de (S.D.M.); anita.thomas@unimedizin-mainz.de (A.T.); axel.haferkamp@unimedizin-mainz.de (A.H.); igor.tsaur@unimedizin-mainz.de (I.T.); olesya.vakhrusheva@unimedizin-mainz.de (O.V.); 2 Department of Urology, Goethe-University, 60590 Frankfurt am Main, Germany; rutzjochen@gmail.com (J.R.); moritz.meiborg@web.de (M.M.); sebastian.maxeiner@unimedizin-mainz.de (S.M.); timothy.grein@kgu.de (T.G.); felix.chun@kgu.de (F.K.-H.C.)

**Keywords:** bladder cancer, mistletoe, growth, proliferation, cell cycling

## Abstract

**Simple Summary:**

Mistletoe extracts are highly popular among cancer patients since they are presumed to counteract oncogenesis and tumor progression. Still, knowledge about mistletoe’s mode of action is limited. The present study analyses the growth and proliferation-blocking properties of mistletoe extracts from four host trees on three bladder cancer cell lines.

**Abstract:**

Extracts of European mistletoe (*Viscum album*) are popular as a complementary treatment for patients with many different cancer types. However, whether these extracts actually block bladder cancer progression remains unknown. The influence of different mistletoe extracts on bladder cancer cell growth and proliferation was investigated by exposing RT112, UMUC3, and TCCSup cells to mistletoe from hawthorn (*Crataegi*), lime trees (*Tiliae*), willow trees (*Salicis*), or poplar trees (*Populi*). The tumor cell growth and proliferation, apoptosis induction, and cell cycle progression were then evaluated. Alterations in integrin α and β subtype expression as well as CD44 standard (CD44s) and CD44 variant (CD44v) expressions were evaluated. Cell cycle-regulating proteins (CDK1 and 2, Cyclin A and B) were also investigated. Blocking and knock-down studies served to correlate protein alterations with cell growth. All extracts significantly down-regulated the growth and proliferation of all bladder cancer cell lines, most strongly in RT112 and UMUC3 cells. Alterations in CD44 expression were not homogeneous but rather depended on the extract and the cell line. Integrin α3 was, likewise, differently modified. Integrin α5 was diminished in RT112 and UMUC3 cells (significantly) and TCCSup (trend) by *Populi* and *Salicis*. *Populi* and *Salicis* arrested UMUC3 in G0/G1 to a similar extent, whereas apoptosis was induced most efficiently by *Salicis*. Examination of cell cycle-regulating proteins revealed down-regulation of CDK1 and 2 and Cyclin A by *Salicis* but down-regulation of CDK2 and Cyclin A by *Populi*. Blocking and knock-down studies pointed to the influence of integrin α5, CD44, and the Cyclin–CDK axis in regulating bladder cancer growth. Mistletoe extracts do block bladder cancer growth in vitro, with the molecular action differing according to the cell line and the host tree of the mistletoe. Integrating mistletoe into a guideline-based treatment regimen might optimize bladder cancer therapy.

## 1. Introduction

Bladder cancer is the most common malignancy of the urinary system, with an estimated 550,000 cases worldwide each year. Most patients (75%) initially present with a localized, non-muscle invasive form. However, the disease inevitably progresses to become muscle-invasive in 20–40% of cases [1]. Although the multimodal treatment strategy has considerably improved in recent years, the prognosis is not good due to high recurrence rates. Once metastasized, the 5-year survival rate is only about 6% [2]. Cisplatin-based chemotherapy is still the standard of care in managing advanced bladder cancer, with a disappointing median overall survival of 13.1 months [3]. Meanwhile, immune checkpoint inhibitors (ICI) have been approved for cisplatin-ineligible patients as a first- or second-line therapy [4]. Nonetheless, only select patients benefit from this treatment and significant side effects and toxicity must be considered, while quality of life and overall survival are not improved [4].

A dissatisfaction with conventional cancer therapy and the wish to actively fight the disease with the hope of a cure has driven many cancer patients to turn to complementary and alternative medicine (CAM) options [5]. Among the plethora of CAM options, extracts of European mistletoe (*Viscum album*) enjoy great popularity, with one-third of patients being reported to use mistletoe, particularly as an injectable prescription drug [6]. Preclinical studies have reported an impact of the extracts on tumor growth, apoptosis, invasion, and immunomodulation, mainly caused by lectins and viscotoxins [7,8]. Experiments on non-small-cell lung cancer cells have provided evidence that the use of mistletoe may not only enhance cisplatin-related effects [9], but may also circumvent cisplatin resistance [10]. This is compelling in regard to bladder carcinoma.

Information related to bladder cancer is sparse and most relevant articles are obsolete. The exposure of mistletoe to a panel of bladder carcinoma cell lines has been shown to suppress tumor proliferation in vitro [11]. This effect has been verified in an orthotopic murine model, documenting a growth blockade of urinary bladder carcinoma following intravesical application of an aqueous mistletoe extract. The formation of multiple metastases, however, was not preventable [12]. In partial contrast, the development of chemically induced urothelial carcinomas in rats could not be reduced when mistletoe extracts were injected subcutaneously [13]. Discrepant effects are also seen when comparing the few clinical trials. The instillation of mistletoe in patients with non-muscle invasive bladder cancer was shown to be safe and well tolerated, with promising efficacy [14]. Based on a retrospective analysis of patients with respectable bladder cancer undergoing high-dosed mistletoe treatment, mistletoe apparently reduced the frequency of tumor recurrence [15]. Accordingly, applying an aqueous mistletoe extract intravesically to patients with superficial urothelial bladder carcinomas led to recurrence rates comparable to controls that had been treated with adjuvant Bacillus Calmette–Guerin [16]. In another study, the adjuvant subcutaneous use of mistletoe lectin after transurethral resection did not affect the time to first recurrence, total number of recurrences, or recurrence-free outcome of patients with superficial bladder cancer [17].

To further examine the potential clinical relevance of mistletoe extracts, an in vitro study has been initiated to explore the growth blocking potential of mistletoe prepared from different host trees on three different bladder cancer cell lines. Besides assessing growth and proliferation, cell cycle-regulating proteins of the CDK (cyclin-dependent kinases) and cyclin families were evaluated. Integrin α and β subtypes, as well as CD44 receptors (standard (CD44s) and variants (CD44v)), were also explored. Integrins are heterodimeric transmembrane glycoprotein signaling receptors composed of an α and β subunit. In mammals, 18 α and 8 β subunits have been identified that impact a wide range of cellular functions including cell migration, growth, proliferation, and apoptosis. CD44 is a non-kinase cell surface transmembrane glycoprotein, largely expressed in the CD44s form, encoded by constant exons. However, post-translational alternative splicing may occur in variant exons to form different CD44v types (CD44v1-10). Along with integrin subtypes, CD44 members are involved in the fate of tumor cells and are associated with bladder cancer size, tumor grade, and recurrence [18].

## 2. Materials and Methods

### 2.1. Cell Lines

Bladder transitional cell carcinoma lines RT112 (grade 2/3) and UMUC3 (grade 3) were purchased from ATCC/LGC Promochem GmbH (Wesel, Germany) and TCCSup (grade 4) from DSMZ (Braunschweig, Germany). All cell lines were grown in Isocove’s Modified Dulbecco’s Medium (IMDM; Gibco/Invitrogen, Karlsruhe, Germany) containing 10% fetal calf serum (FCS), 2% glutaMAX, and 1% penicillin/streptomycin (all: Gibco/Invitrogen) in a humidified 5% CO_2_ incubator. Data were collected from cultures with passages below 24.

### 2.2. Mistletoe Extracts

Samples of mistletoe plants (*Viscum album* L.) were collected from four host tree species, namely hawthorn (*Crataegi*), lime trees (*Tiliae*), willow trees (*Salicis*), and poplar trees (*Populi*), according to the official production protocol described in the German Homoeopathic Pharmacopoeia (GHP) version 38 [19]. The mistletoe extracts are identified by their host trees (*Cratagei*, *Tiliae*, *Salicis*, *Populi*). The fresh mistletoes were cultivated on and harvested from WALA Heilmittel GmbH (Bad Boll/Eckwälden, Germany) dedicated cultivation areas [20]. The mother tincture was diluted 1:8000 up to 1:3.2 × 10^6^ (*Tiliae*) or 1:8 × 10^6^ (*Crataegi, Salicis, Populi*). Isotonic solvent or cell culture medium alone was used as control.

### 2.3. Tumor Cell Growth

Cell growth was analyzed by a 3-(4,5-dimethylthiazol-2-yl)-2,5-diphenyltetrazolium bromide (MTT) dye reduction assay (Roche Diagnostics, Penzberg, Germany). Tumor cells were detached from the culture flask by enzymatic treatment using accutase (PAA Laboratories, Cölbe, Germany). Each well of a 96-well plate was then filled with 5,000 cells and exposed to a mistletoe extract or control medium without extract (each in triplicate). Cells were allowed to settle down to establish firm adhesion contact and to recover from enzymatic treatment overnight. Tumor cell number was then counted after 72 h. After 24, 48, and 72 h incubation, 10 µL MTT (0.5 mg/mL) was added for an additional 4 h. Cells were then lysed overnight in solubilization buffer (10% SDS in 0.01 M HCl) at 37 °C, 5% CO_2_. A microplate reader was used to detect absorbance at 550 nm (Tecan Infinite M200, Männedorf, Switzerland). To convert the absorbance into an absolute cell number, a standard curve was prepared. In total, 2500 to 160,000 cells/well were seeded onto a 96-well plate in triplicate, subsequently lysed after metabolization of the MTT, and absorbance was measured. Data were presented graphically as dose–response kinetics, after offsetting with a standard curve and normalization to 24 h.

### 2.4. Tumor Cell Proliferation

Quantification of cell proliferation was performed with a 5-bromo-2′-deoxyuridine (BrdU)-specific antibody (BrdU cell proliferation enzyme-linked immunosorbent assay (ELISA) kit; Calbiochem/Merck Biosciences, Darmstadt, Germany). Then, 50 μL tumor cell suspension (10^5^ cells/mL) was added to 96-well microtiter plates and then incubated with 20 μL of BrdU labeling solution for 24 h. Cells were then fixed and detected using anti-BrdU mAb, according to the manufacturer’s instructions. Absorbance was measured at 450 nm using a microplate ELISA reader (Tecan Infinite M200, Männedorf, Switzerland). Values were expressed as percentage compared to untreated controls (set to 100%).

### 2.5. Integrin and CD44 Surface Expression

Bladder cancer cells were enzymatically detached from the culture flasks (Accutase^®^; PAA Laboratories GmbH, Pasching, Austria), followed by washing with blocking solution (PBS, 0.5% BSA) and incubation with phycoerythrin (PE) conjugated monoclonal antibodies (1 h at 4 °C) directed against the following integrin subtypes: anti-integrin α1 (IgG1; clone SR84), anti-integrin α2 (IgG2a; clone 12F1-H6), anti-integrin α3 (IgG1; clone C3II.1), anti-integrin α4 (IgG1; clone 9F10), anti-integrin α5 (IgG1; clone IIA1), anti-integrin α6 (IgG2a; clone GoH3), anti-integrin β1 (IgG1; clone MAR4), anti-integrin β3 (IgG1; clone VI-PL2), and anti-integrin β4 (IgG2a; clone 439-9B; all: BD Biosciences, San Jose, CA, USA). Mouse IgG1-PE (MOPC-21), mouse IgG2a-PE (G155-178), or rat IgG2b-PE (R35-38; all: BD Biosciences) were used as isotype controls. To investigate CD44 standard (CD44s) or CD44 variants (CD44v) expression, all antibodies, anti-CD44s (clone SFF-2), anti-CD44v3 (clone VFF-327v3), anti-CD44v4 (clone VFF-11), anti-CD44v5 (clone VFF-8), anti-CD44v6 (clone VFF-7), and anti-CD44v7 (clone VFF-9; all: eBioscience, ThermoFisher), were conjugated to allophycocyanin (APC) using the Lightning-Link APC Conjugation Kit (eBioscience, ThermoFisher, Darmstadt, Germany) and then reacted with bladder cancer cells for 60 min at 4 °C. Mouse IgG1, K (clone P3.6.2.8.1; ThermoFisher, Dreieich, Germany), coupled to APC, acted as the isotype control. Analysis was performed by an FACSCalibur (BD Biosciences; FL2-H (integrins) or FL4-H (CD44s/CD44v) (log) channel histogram analysis; 1 × 10^4^ cells per scan). Fluorescence values were expressed as mean fluorescence units.

### 2.6. Apoptosis

To evaluate apoptotic events, the annexin V-FITC Apoptosis Detection kit (BD Pharmingen, Heidelberg, Germany) was applied. After incubation with mistletoe extracts, tumor cells were detached, washed twice with PBS, and then reacted with 5 μL of anti-annexin V-FITC antibodies and 5 μL of DNA-intercalating dye, propidium iodide (PI), under light protection for 15 min. Samples were then subjected to an FACScalibur (BD Biosciences; 1 × 10^4^ cells per scan) and analyzed with CellQuest software version 5.1 (BD Biosciences). Possible cytotoxic effects of the mistletoe extracts were explored by staining with cell non-permeable trypan blue (Gibco/Invitrogen, Darmstadt, Germany).

### 2.7. Cell Cycling

Bladder cancer cell cycle progression after treatment with mistletoe extracts (versus untreated controls) was analyzed using CycleTest™ Plus DNA Reagent Kit (Becton Dickinson, Heidelberg, Germany) according to the manufacturer’s instructions. Fluorescence was measured with a flow cytometer FACSCalibur (BD Biosciences, 1 × 10^4^ cells per sample). Data acquisition and calculation of cell cycle distribution were carried out using CellQuest version 5.1 and ModFit software version 3.3 (BD Biosciences). The number of gated cells in the G1, G2/M, or S phase is given in percent.

### 2.8. Western Blot Analysis

Bladder cancer protein lysates were separated on a 7–12% polyacrylamide gel (depending on the protein size) via electrophoresis at 100 V for 90 min. The proteins were then transferred to nitrocellulose membranes (1 h, 100 V). Membranes were blocked with skim milk powder for 1 h and then incubated overnight with the following monoclonal antibodies: CDK1/Cdc2 (IgG1, clone 1), CDK2 (IgG2a, clone 55), Cyclin A (IgG1, clone 25), and Cyclin B (IgG1, clone 18; all from BD Biosciences). Secondary HRP-conjugated goat anti-mouse IgG and goat anti-rabbit IgG (both: Cell Signaling) antibodies were used. Membranes were exposed to luminol-based chemiluminescent substrate (ECL) from Amersham/GE Healthcare, München, Germany, and then evaluated by Fusion FX7 (Peqlab, Erlangen, Germany) to illustrate protein expression. Expression of all tested proteins was normalized to the expression of β-actin (Cell Signaling). GIMP 2.8.20 software (https://www.gimp.org/downloads/; assessed on 8 January 2023) was utilized to estimate the pixel intensity of individual bands.

### 2.9. Blocking Studies

UMUC3 tumor cells were pre-incubated with function-blocking antibodies, anti-integrin α3 or anti-integrin α5 mouse mAb (10 µg/mL each; all from Merck Millipore, Darmstadt, Germany), and then subjected to the MTT assay. To investigate integrin-related signaling, UMUC3 cells were treated with 10 µM of the FAK inhibitor defactinib (Biozol, Eching, Germany) for 24 h. CD44s was blocked with a function-associated monoclonal antibody (clone SFF-2) which recognizes human standard CD44 but does not distinguish between the splice variants.

### 2.10. Knockdown Studies

Transfection of UMUC3 cells with small interfering RNA (siRNA) was performed using HiPerFect Transfection reagent (Qiagen, Hilden, Germany). siRNAs were directed against CDK1 (gene ID: 983, target sequence: AAGGGGTTCCTAGTACTGCAA) or CDK2 (gene ID: 1017, target sequence: AGGTGGTGGCGCTTAAGAAAA) or Cyclin A (gene ID: 890, target sequence: GCCAGCTGTCAGGATAATAAA; all purchased from Qiagen). siRNA was mixed with transfection reagent in a 1:6 ratio and incubated for 24 h with 3 × 10^5^ UMUC3 cells. Untreated cells and control cells reacting with AllStars Negative Control siRNA (Qiagen) served as controls. The protein expression level was verified by Western blotting. Tumor cell number of the specimen was then analyzed by the MTT assay.

### 2.11. Statistics

The mean ± SD was calculated. Graphs were prepared using SigmaPlot 11 (SYSTAT Software version 13.2, San Jose, CA, USA). To exclude coincidence, all experiments were repeated three to five times. Statistical significance was evaluated with the “Student’s *t*-test”. *p* < 0.05 was considered significant.

## 3. Results

### 3.1. Influence of Mistletoe Extracts on Tumor Growth and Proliferation

**Figure 1:**
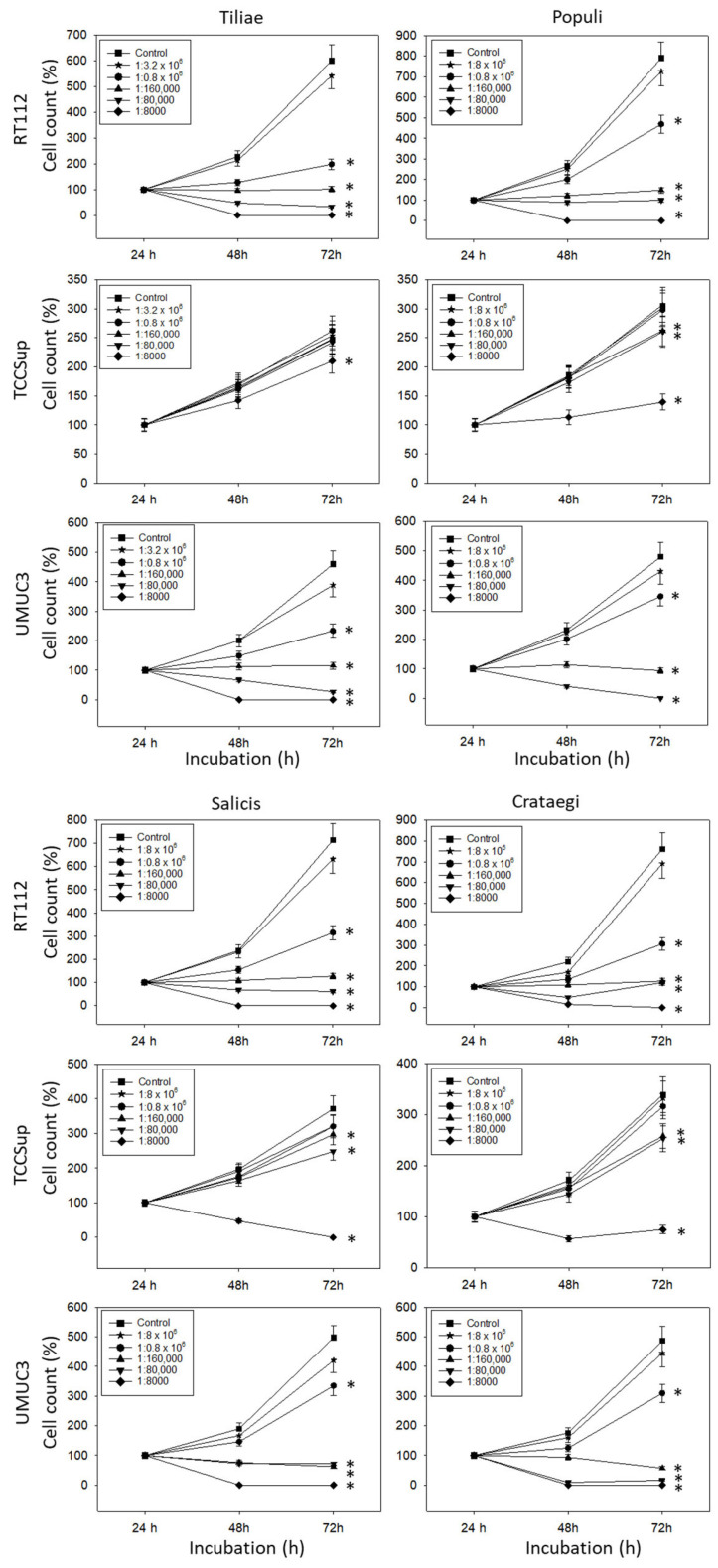
Influence of increasing concentrations of four mistletoe extracts (*Tiliae, Populi, Salicis, Crataegi*) on the growth of RT112, TCCSup, and UMUC3 bladder cancer cell lines. The cell number was evaluated after 24, 48, and 72 h by the MTT assay, whereby 24 h values were set to 100%. “Control” indicates medium control. Error bars indicate standard deviation. Experiments were performed three times. * indicates significant difference to the corresponding control.

All mistletoe extracts significantly down-regulated the growth of all tested bladder cancer cell lines (Figure 1). The effects were most apparent with RT112 and UMUC3 cells, where a 1:0.8 × 10^6^ dilution already induced a marked cell loss during the 72 h observation period. The application of 1:160,000 dilutions and lower induced a near complete stop of RT112 and UMUC3 tumor growth. Higher concentrations were necessary to suppress the growth of TCCSup cells. No differences were seen in cell growth activity in the presence of isotonic solvent or cell culture medium alone (solvent control values are not included in the figure).

**Figure 2:**
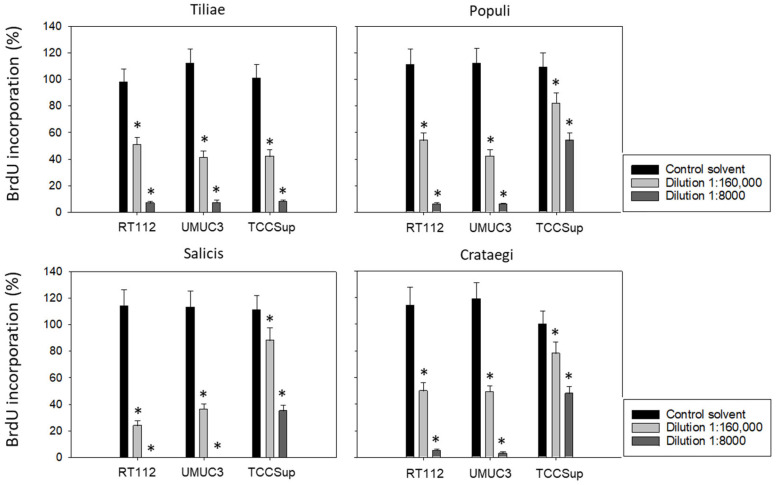
Influence of four mistletoe extracts (*Tiliae, Populi, Salicis, Crataegi*) on the proliferation of RT112, UMUC3, and TCCSup cells. Proliferative activity was analyzed by the BrdU incorporation assay following 48 h mistletoe incubation. Error bars indicate standard deviation. * indicates a significant difference to controls (tumor cells exposed to culture medium alone) set to 100%. *n* = 3.

Subsequent studies were conducted with 1:8000 and 1:160,000 dilutions of the mistletoe extracts to evaluate BrdU uptake in treated versus non-treated tumor cells. All mistletoe extracts reduced BrdU incorporation significantly. However, the response of TCCSup to the extracts from *Populi*, *Salicis*, and *Crataegi* was much less pronounced compared to that of RT112 and UMUC3 (Figure 2). Employing the 1:8000 dilution, *Populi* and *Crategi* reduced BrdU incorporation by about 90% in RT112 and UMUC3 but by only 50% in TCCSup. BrdU incorporation was completely blocked in RT112 and UMUC3 cells in the presence of *Salicis*, whereas the same extract diminished BrdU in TCCSup by only 65%. Distinct differences were also seen when the tumor cells were exposed to *Populi, Salicis*, and *Crataegi* at a 1:160,000 dilution with a suppression of 75% in RT112 and 65% in UMUC3, in contrast to only 12% suppression in TCCSup.

### 3.2. Mistletoe Extracts Alter Integrin α and β and CD44s and CD44v Expression

**Figure 3:**
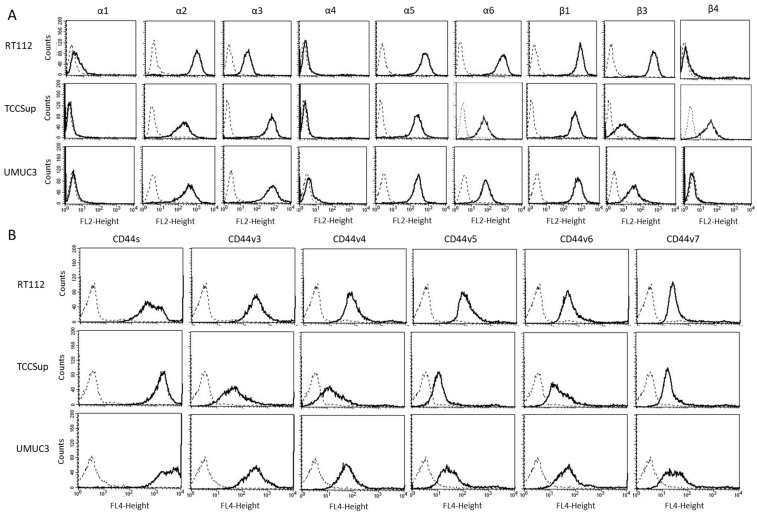
(**A**) surface expression of α and β integrins. (**B**) surface expression of CD44 standard (CD44s) and CD44 variants v3, v4, v5, v6, and v7. The abscissa shows the relative logarithmic distribution of the relative fluorescence intensity, the ordinate shows cell number. The solid line indicates specific fluorescence, the dashed shows the IgG1 isotype control. One representative figure from *n* = 3.

**Figure 4:**
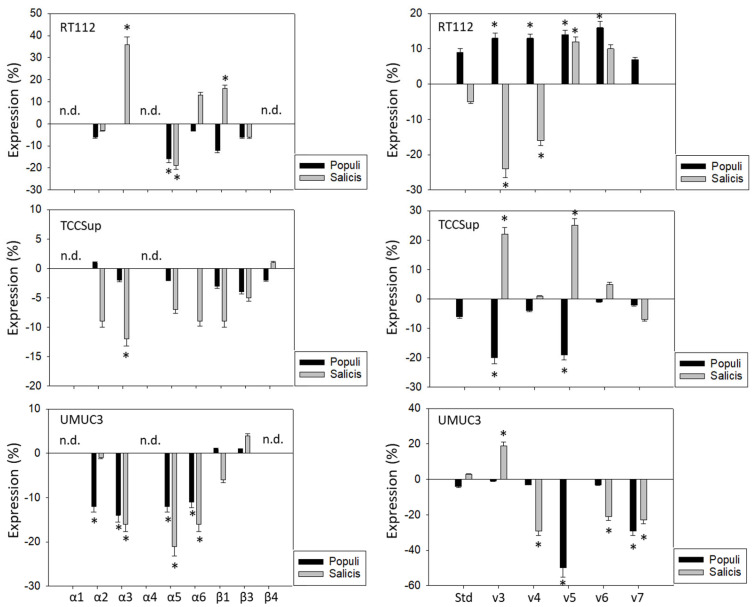
Influence of *Populi* and *Salicis* mistletoe extracts (Dilution 1:160,000) on the integrin (**left**) or CD44 standard (Std) and CD44 variants v3–v7 expression profile (**right**) of RT112, TCCSup, and UMUC3 cells. The untreated control is set to 0. Values are means ± SD, *n* = 4. Error bars indicate standard deviation. * indicates significant difference to controls. n.d.= not detectable.

The expression level of various integrins (Figure 3) on untreated RT112, TCCSup, and UMUC3 cells show that integrins α1 and α4 were not expressed on any of these cell lines while α2, α5, and β1 were strongly expressed. β3 was highly expressed on RT112 cells, but moderately expressed on TCCSup and UMUC3. α3 was strongly expressed on TCCSup and UMUC3 cells but to a lesser extent on RT112. α6 was strongly detectable on RT112, but only moderately present on TCCSup and UMUC3 cells. Integrin β4 was only expressed on TCCSup. CD44 receptors (Figure 4) were identified on all cell lines with slight differences among them. CD44v3-v6 was detected on TCCSup to a lesser extent, compared to RT112 and UMUC3. The surface expression of CD44s and CD44v7, however, was similar in all three cell lines.

*Populi* and *Salicis* extracts at a dilution of 1:160,000, which had been shown to cause the strongest inhibitory effects on tumor growth and proliferation on the tumor cells, were chosen to evaluate their effects on integrins and CD44 in all three cell lines. The influence of the two mistletoe extracts on the three cell lines was not the same. The extract from *Salicis* up-regulated integrin α3 on RT112 (+36%) but down-regulated α3 on TCCSup (−12%) and UMUC3 (−16%) (Figure 4, left). The extract from *Populi* also down-regulated integrin α3 on UMUC3 cells (−14%). The subtype α5 was reduced on RT112 and UMUC3, but not on TCCSup, by both *Populi* (mean reduction on RT112 and UMUC3: −15%) and *Salicis* (mean reduction on RT112 and UMUC3: −20%). Both extracts suppressed α6 on UMUC3 (*Populi*: −11%, *Salicis*: −16%), but not on RT112 and TCCSup. Similarly, α2 was down-regulated by *Populi* on UMUC3 (−12%) but not on RT112 and TCCSup. The dissimilar behavior of the tumor cells also became evident in regard to CD44 alterations (Figure 4, right). Treatment with *Populi* extract enhanced CD44v3-v6 on RT112, whereas *Salicis* enhanced CD44v5 (+12%) but diminished the expression of CD44v3 (−25%) and CD44v4 (−16%) on RT112. Opposite effects were also apparent on TCCSupp, with CD44v3 and CD44v5 being enhanced by *Salicis* (v3: +22%, v5: +25%) but reduced by *Populi* (both: −20%). The extract from *Populi* suppressed the expression of CD44v5 (-50%) and CD44v7 (−30%) on UMUC3, while *Salicis* extract suppressed CD44v4 (−30%), CD44v6 (−20%), and CD44v7 (−20%), but elevated CD44v3 (+20%). Since UMUC3 exhibited the highest sensitivity to both extracts, *Populi* and *Salicis*, this cell line was used for further studies.

### 3.3. Influence of Mistletoe Extracts on Apoptosis and Cell Cycling

**Figure 5:**
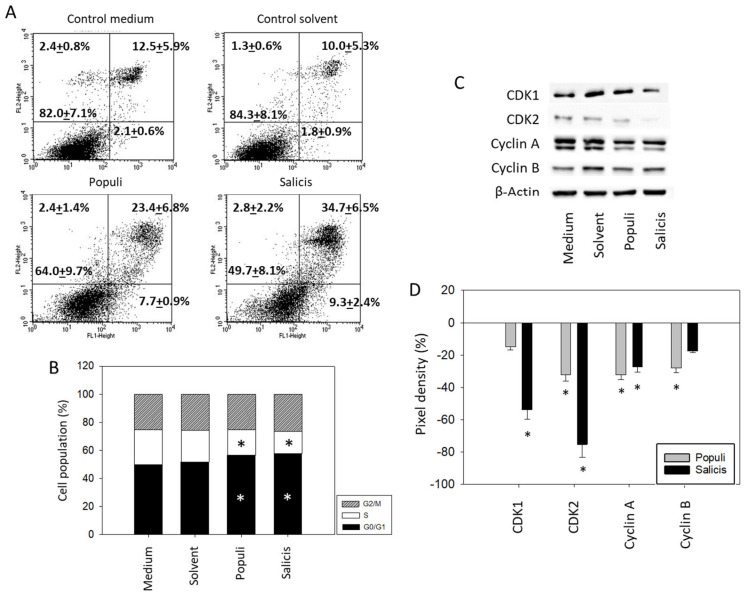
Apoptosis and cell cycling. UMUC3 cells were treated with mistletoe from *Populi* or *Salicis*. Controls remained untreated (Medium) or were treated with solvent alone (Solvent). (**A**) apoptosis induction (dilution of 1:8000). Upper left quadrants show the percentage of cells in necrosis, upper right quadrants in late apoptosis, lower right quadrants in early apoptosis, and lower left quadrants vital cells (percentage values are means ± SD from n = 3). (**B**) cell cycle distribution evaluated after 24 h incubation. * indicates significant difference to the controls. (**C**) CDK1, CDK2, Cyclin A, and Cyclin B protein expression in UMUC3 cells detected by Western blotting (Full pictures of the Western blots are presented in the Supplementary Materials file). (**D**) presentation of the pixel density data. The results are given in percentage and related to CDK1, CDK2, Cyclin A, and Cyclin B expression in the untreated controls. (**B**–**D**) depict a dilution of 1:160,000. * indicates significant difference to controls (*n* = 3).

Treatment with extracts from *Populi* and *Salicis* at dilutions of 1:8000 strongly elevated the number of UMUC3 cells undergoing early and late apoptosis, whereas necrosis remained unchanged (*Salicis* > *Populi*; Figure 5A). Dilutions of 1:160,000 did not induce signs of apoptosis. A cell cycle analysis in the presence of dilutions of 1:160,000 showed a significant increase in G0/G1 phase cells (Medium control: 51.7 ± 4.4%, *Populi*: 58.1 ± 4.8%, *Salicis*: 59.7 ± 6.6%), accompanied by a loss of S phase cells (Medium control: 16.7 ± 2.9%, *Populi*: 13.1 ± 4.0%, *Salicis*: 12.2 ± 1.6%) (Figure 5B). Cell cycle-regulating proteins were evaluated (Figure 5C,D, Supplementary Figure S1), whereby CDK1 was reduced by *Salicis* and Cyclin B was reduced by *Populi*. CDK2 (*Salicis* > *Populi*) and Cyclin A were diminished by both extracts.

### 3.4. Blocking Studies

**Figure 6:**
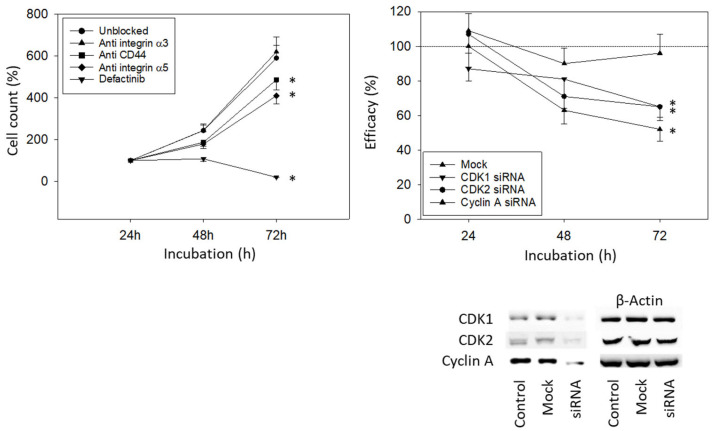
Blocking and knock-down studies. (**Left**) UMUC3 cell number in response to integrin blockade (Anti integrin α3, anti integrin α5), to CD44s blockade (Anti CD44), and to blockade with defactinib (Defactinib). Untreated cells served as controls. Cell number was evaluated after 24, 48, and 72 h by the MTT assay (*n* = 3). (**Right**) cell growth of UMUC3 treated with a CDK1-, CDK2-, or Cyclin A-specific siRNA, evaluated by the MTT assay. The Western blot on lower right depicts reduction in CDK1 and CDK2 by siRNA knock down (Supplementary Figure S1). One representative of three separate experiments is shown. All values are related to the untreated controls set to 100%. Mock indicates scrambled controls. * indicates significant difference to the untreated (unblocked) control or to cells not treated with siRNA.

To investigate how the integrin subtypes α3 and α5 are involved in bladder cancer cell growth, UMUC3 cells were blocked with integrin function-blocking antibodies. Blocking the integrin α5, but not α3, was associated with a significant reduction in UMUC3 growth. Defactinib was used to block focal adhesion kinase (FAK), which is involved in integrin downstream signaling. When the integrin downstream signaling was altered by defactinib, a known focal adhesion kinase (FAK) inhibitor, an even stronger decrease in cell growth was documented, compared to the integrin α5 blockade (Figure 6, left). Blocking CD44s was associated with reduced growth activity as well (Figure 6, left). Knocking down CDK1, CDK2, and Cyclin A significantly reduced the tumor cell number (Figure 6, right).

## 4. Discussion

Anti-tumor properties have previously been associated with the origin and preparation of mistletoe extracts [21]. In the present study, the *Tiliae* extract was found to be superior to the *Crataegi*, *Salicis*, or *Populi* extracts in suppressing growth in UMUC3 and RT112 cells. The difference was particularly apparent when the extracts were applied at a high dilution of 1:0.8 × 10^6^. TCCSup cells did not respond to the *Tiliae* extract diluted 1:0.8 × 10^6^, but more concentrated extracts from all four mistletoe specimens altered tumor cell growth and proliferation similarly. Accordingly, exposing bladder cancer cells to seven different mistletoe extracts revealed that *Tiliae* and *Crataegi* exhibited the strongest cytotoxic effects [22]. In a lung carcinoma cell model, *Viscum album* preparations from *Tiliae* and *Salicis* host trees most strongly inhibited proliferation, but strong effects were also found with *Crataegi* and *Populi* mistletoe [19]. Exposing lymphoblastic leukemia or sarcoma cells to *Viscum album subsp. album* growing on *Malus domestica*, *Quercus robur*, and *Ulmus carpinifolia* altered cell viability to a similar extent. However, distinct differences were recorded compared to cells treated with *Viscum album* from the subspecies *austriacum* and *abietis* [23]. The reason for these differences is not clear. Holandino et al. surmised that the viscotoxin content might be related to the potency of the extracts [23]. Others consider the overall chemical composition, including lectin and viscotoxin cross communication, to be responsible for the anti-tumor activity [19]. Thus, the mistletoe species or host tree does seem to determine the cytotoxic efficacy.

Other factors to be considered in regard to mistletoe efficacy include the tumor type as well as the tumor differentiation status. The present investigation was conducted with three different tumor cell lines and TCCSup cells were found to be less sensitive to the mistletoe extracts than UMUC3 and RT112 cells. With a similar approach, the cell growth of UMUC3 cells was blocked more potently by *Viscum album Tiliae* than for TCCSup cells [22], and two further studies utilizing *Viscum album* preparations reported different IC_50_ values with TCCSup > UMUC3 [11,21]. RT112 is a grade 2/3, UMUC3 a grade 3, and TCCsup a grade 4 type. Speculatively, low-grade bladder cancer might particularly benefit from mistletoe. Although TCCSup cell growth in our study was poorly inhibited by *Viscum album Tiliae*, BrdU incorporation in this cell line was comparably diminished to that in RT112 and UMUC3 cells.

The different tumor cell lines displayed different integrin receptor expressions. The subtype β4 was strongly present on the TCCSup cell membrane but not detectable on RT112 and UMUC3, and the subtype α3 was only slightly expressed on RT112, but considerably expressed on UMUC3 and TCCSup cells. In keeping with the differentiated cell-specific integrin expression, integrin alterations induced by mistletoe treatment were also different. The most prominent discrepancy was seen for integrin α3 being up-regulated on RT112 but down-regulated on UMUC3 and TCCSup cells. This type of anomaly has been observed previously, whereby an inverse α3 response of TCCsup and UMUC3 compared to RT112 cells was observed when treated with the histone deacetylase inhibitor valproic acid [24]. The authors speculated that the initial integrin composition might determine the success of therapeutic intervention.

An association between integrin α3 down-regulation and the inhibition of clonogenic growth, tumor cell migration, and invasion has previously been reported [25]. Another study indicates differences between RT112 and UMUC3, whereby blocking the α3 function reduced UMUC3 chemotaxis but enhanced chemotaxis in RT112 cells [26,27]. Divergent integrin-guided behavior in several tumor sublines has additionally been reported with opposing effects of α3 on HCV29, compared to T24 and Hu456 bladder cancer cells [28]. Knocking down integrin α3 in T24 cells by two different small interfering integrin α3 types significantly suppressed migration and invasion, independent of the type used. However, tumor proliferation was only slightly influenced by one type, whereas the other type did not induce any alteration on tumor growth [29]. A similar scenario may hold true in our model, since blocking integrin α3 with monoclonal antibodies also did not change the growth activity of UMUC3 cells in our experiments. However, since mistletoe extract-induced blockage of integrin α3 does reduce UMUC3 motility [26], mistletoe may still exert an inhibitory effect on the UMUC3 bladder cancer cell line.

The integrin α5 was strongly diminished by *Viscum album Salicis* and *Populi* in both RT112 and UMUC3, and moderately, though not significantly, down-regulated on TCCSup. Blocking studies on UMUC3 cells indicate a growth-suppressive function of α5, indicating that this integrin member might play a decisive role in bladder cancer growth control. Its expression has been shown to affect bladder cancer cell phenotypes by facilitating cell viability, migration, and invasion [30]. A high integrin α5 level in the cytomembrane of tumor cells has been related to shorter overall survival in bladder cancer patients [31]. Mistletoe extracts, therefore, may contribute to slowing bladder cancer growth and proliferation by reducing integrin α5 expression. This assumption is supported by defactinib blocking experiments showing a close association between FAK and bladder cancer growth. FAK integrin α5 communication has been shown to promote tumor cell proliferation [32], pointing to mistletoe extracts from *Salicis* and *Populi* being FAK integrin α5 inhibitors.

An inhomogeneous response from the bladder cancer cell lines to the mistletoe extracts was also apparent in regard to CD44 expression. In a panel of 13 bladder cancer cell lines with different degrees of differentiation, there was an absence of CD44v5 in high-grade cell lines, while CD44v6 expression did not show any association with tumor grade or stage [33]. Silencing CD44v3 has been reported to promote tumor progression [34], whereas the opposite has been shown by Toma et al., documenting a significant association between a loss of CD44v3 and a short recurrence-free interval [35]. This finding differs considerably from another study where a high CD44v6 expression was associated with tumor recurrence [18]. It has been reported that shorter splicing codes characterized by high CD44s levels could be key factors driving bladder cancer progression and are associated with a worse prognosis [36]. A similar hypothesis has been proposed by Zhang et al., who have presented evidence that switching CD44v to CD44s may determine tumor cell aggressiveness [37]. Although CD44s’s expression was not altered by the mistletoe extracts after 24 h exposure in our study, blocking CD44s significantly suppressed tumor cell growth. Experiments with prostate cancer cells show that incubation with tumor-suppressive drugs over a longer period (> 48h) might be necessary to trigger a strong up-regulation of CD44v surface expression [38]. Whether prolonged exposure to mistletoe might be required to induce a CD44 switch from CD44s to CD44v in the bladder cancer cell model requires further investigation.

Cell cycling, cell cycle-related proteins, and apoptosis in UMUC3 cells were evaluated after exposure to *Viscum album Salicis* or *Populi*. Considerable increases in both early and late apoptosis were apparent, with *Salicis* being superior to *Populi*. The number of cells driven to late apoptosis was higher than the number of cells driven to early apoptosis. Apoptosis induction following treatment with *Viscum album* L. grown on oak (*Quercus*) has been shown in T24, TCCSup, J82, and UMUC3 cells [11]. Apoptotic events have also been observed in further solid tumors such as lung [39], breast [40], and colon cancer [41], but the cytotoxic potential of mistletoe varies according to the tumor type. TCCsup cells were less sensitive to mistletoe, compared to J82 and T24 bladder cancer cells [11]. Harmsma et al. found low and high responders with cell lines derived from colorectal carcinoma being less sensitive to apoptosis induction by mistletoe, compared to lung and breast cancer cells. Different modes of action were also noted for the extracts from the different host trees. Mistletoe from *Malus* induced apoptosis via the activation of the death receptor, mistletoe from *Quercus* activated the mitochondrial pathway, whereas mistletoe *Pinus* was found to mainly induce necrosis [42].

Our data point to higher concentrations of the mistletoe extracts to induce apoptosis, compared to the concentration necessary for cell growth suppression. Presumably, growth-suppressing effects dominate over the apoptosis-inducing action’s of (at least) *Salicis* or *Populi.* In fact, inhibited bladder cancer growth may not always be due to apoptosis induction. *Viscum album Quercus* strongly suppressed the growth of four bladder carcinoma cell lines, while two of them underwent apoptosis only to a minor degree [11]. We have observed a similar phenomenon. The mistletoe extract from *Salicis* was much more potent than *Populi* in inducing apoptosis in UMUC3 cells, although both compounds suppressed UMUC3 growth to a similar extent. This mechanistic difference accords with the observation that both *Viscum album Populi* and *Viscum album Salicis* arrested UMUC3 cells in G0/G1, whereas cell cycle-related signaling proteins were altered differently. The mistletoe from *Salicis* considerably diminished CDK1 and 2, whereas Cyclin B was suppressed by *Populi* but not by *Salicis*. Different molecular mechanisms of action may therefore be responsible for effectively down-regulating tumor growth through the mistletoe extracts. The present study and former experiments on bladder cancer cells revealed that CDK1 and 2 and their corresponding binding partners, Cyclin B and A, serve as crucial growth mediators [43]. Mistletoe from *Salicis* acts on UMUC3 growth via CDK1 and 2 and Cyclin A, and mistletoe from *Populi* via CDK2, and Cyclin A and B. Additional evidence indicates that CDK1 inversely correlates with apoptosis induction [44]. A loss of CDK1 was only seen when UMUC3 cells were exposed to mistletoe from *Salicis*, which might explain why apoptosis was most prominent in this cell line. Cyclin A in combination with CDK1 initiates the switch from the G2 to M phase, but, in combination with CDK2, initiates S phase progression. Cyclin B with CDK1 is significantly involved in M phase progress. Although the cell response is ambiguous, the down-regulation of the tested cell cycle proteins after mistletoe treatment fits well with the detected G0/G1 arrest.

Apoptosis induction and cell cycle arrest have also been observed in other tumor entities. Similar to our observations, mistletoe (*Viscum album var*) obstructed the cell cycle of hepatocarcinoma cells via G1 phase arrest, accompanied by down-regulated CDK2 [45]. *Viscum album Quercus* lowered the number of lung and breast cancer S phase cells (but not G0/G1 phase cells) and increased late apoptosis [42]. Mistletoe from *Malus domestica* instead induced a significant G2/M accumulation of breast cancer cells and diminished the proportion of S phase cells [46]. Kleinsimon et al. point to the potential role of the p53 status of the cells and presented evidence that mistletoe extracts induced G1 arrest in p53 wild-type and null-mutant, but S phase arrest in p53 mutant osteosarcoma cell lines [47].

The applicability of mistletoe for breast and gynecological cancer patients when combined with targeted therapies has been shown [48]. A mixed-phase pilot placebo-controlled, double-blind randomized controlled trial of mistletoe therapy in patients with breast cancer has been started, of which the standard treatment plan includes chemotherapy with or without radiotherapy [49]. A phase I trial on patients with solid tumors progressing on chemotherapy has demonstrated disease control and improved quality of life with intravenously administered mistletoe [50]. Accordingly, integrating mistletoe into standard treatment regimens for bladder cancer could, likewise, improve long-term outcomes and should be investigated.

Side effects from mistletoe, including local skin reactions at the injection site and systemic reactions such as fever, fatigue, rash, and flu-like symptoms [51,52], have been documented. When mistletoe was applied intravenously to cancer patients, treatment had to be discontinued due to toxicity in 15% of cases (grade 3 fatigue, grade 3 alanine aminotransferase elevation, or grade 3 dyspnea/flank pain) [50]. Hence, establishing appropriate dosage is important and requires further study. More studies should be directed towards examining cell signaling processes in other bladder cancer cell lines, not only to verify our observations but also to exclude undesired events or undesired protein–protein cross communication that could reduce the tumor cell’s response to mistletoe or even re-activate oncoproteins.

## 5. Conclusions

Mistletoe extracts derived from different host trees significantly suppressed the growth of different bladder cancer cells and induced apoptosis in vitro. This is the first report identifying integrin adhesion receptors and CD44 as targets of mistletoe extracts relevant for modulating the growth and proliferation of bladder cancer cells. The integrin α5 was strongly diminished by *Salicis* and *Populi* on both RT112 and UMUC3, and was moderately down-regulated on TCCSup. Receptor alterations due to the influence of mistletoe were not all the same but rather depended on the origin of the extract and the tumor cell type. The same was true with respect to the Cyclin–CDK axis, which was also disparately targeted by mistletoe extracts. Mistletoe from *Salicis* specifically targeted CDK1 and 2, and Cyclin A, whereas mistletoe from *Populi* acted via CDK2 and Cyclin A and B. Low-grade bladder cancer appears to be more sensitive to mistletoe extracts than high-grade bladder cancer.

## Data Availability

The data presented in this study are available in this article (and Supplementary Materials).

## Supplementary Materials

The following supporting information can be downloaded at: https://www.mdpi.com/article/10.3390/cancers15194849/s1, Figure S1: Western blots.
