# Supplementary figures and images for: Mistletoe Extracts from Different Host Trees Disparately Inhibit Bladder Cancer Cell Growth and Proliferation
Source: Cancers (Basel). 2023 Oct 4;15(19):4849. doi: 10.3390/cancers15194849 (PMC10571756; doi:10.3390/cancers15194849)

S1: Western Blots

Figure 5C

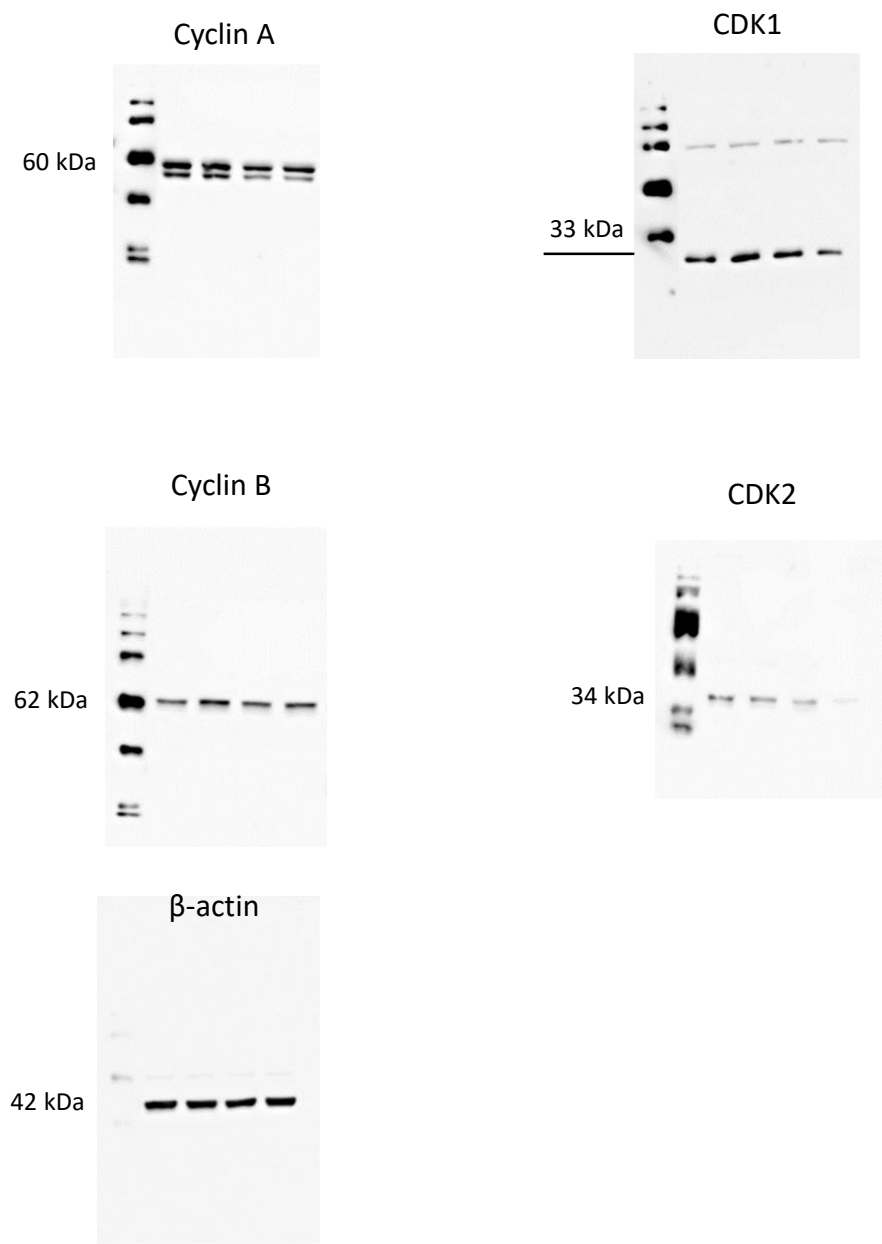

Figure 6

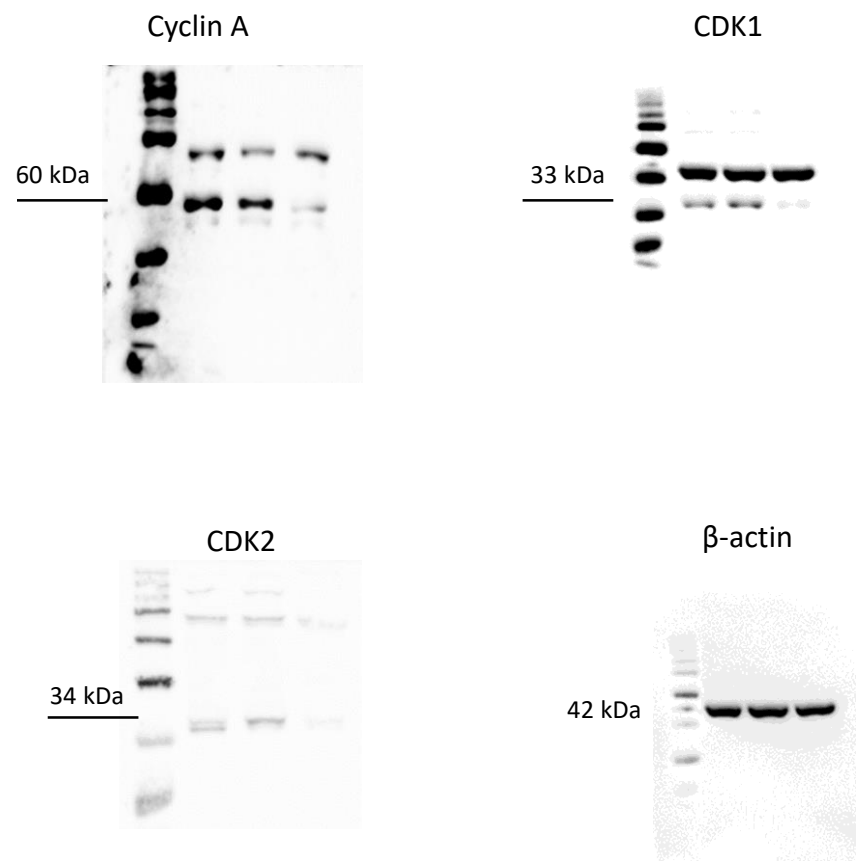

Supplement: Supplementary file 1 [file cancers-15-04849-s001.zip › cancers-2638486-supplementary.pdf]
